# Supplementary material for: Ezh2 emerges as an epigenetic checkpoint regulator during monocyte differentiation limiting cardiac dysfunction post-MI
Source: Nat Commun. 2023 Jul 25;14:4461. doi: 10.1038/s41467-023-40186-0 (PMC10368741; doi:10.1038/s41467-023-40186-0)
Supplement: Supplementary file 1 — Supplementary Information [file 41467_2023_40186_MOESM1_ESM.pdf]

Supplementary information

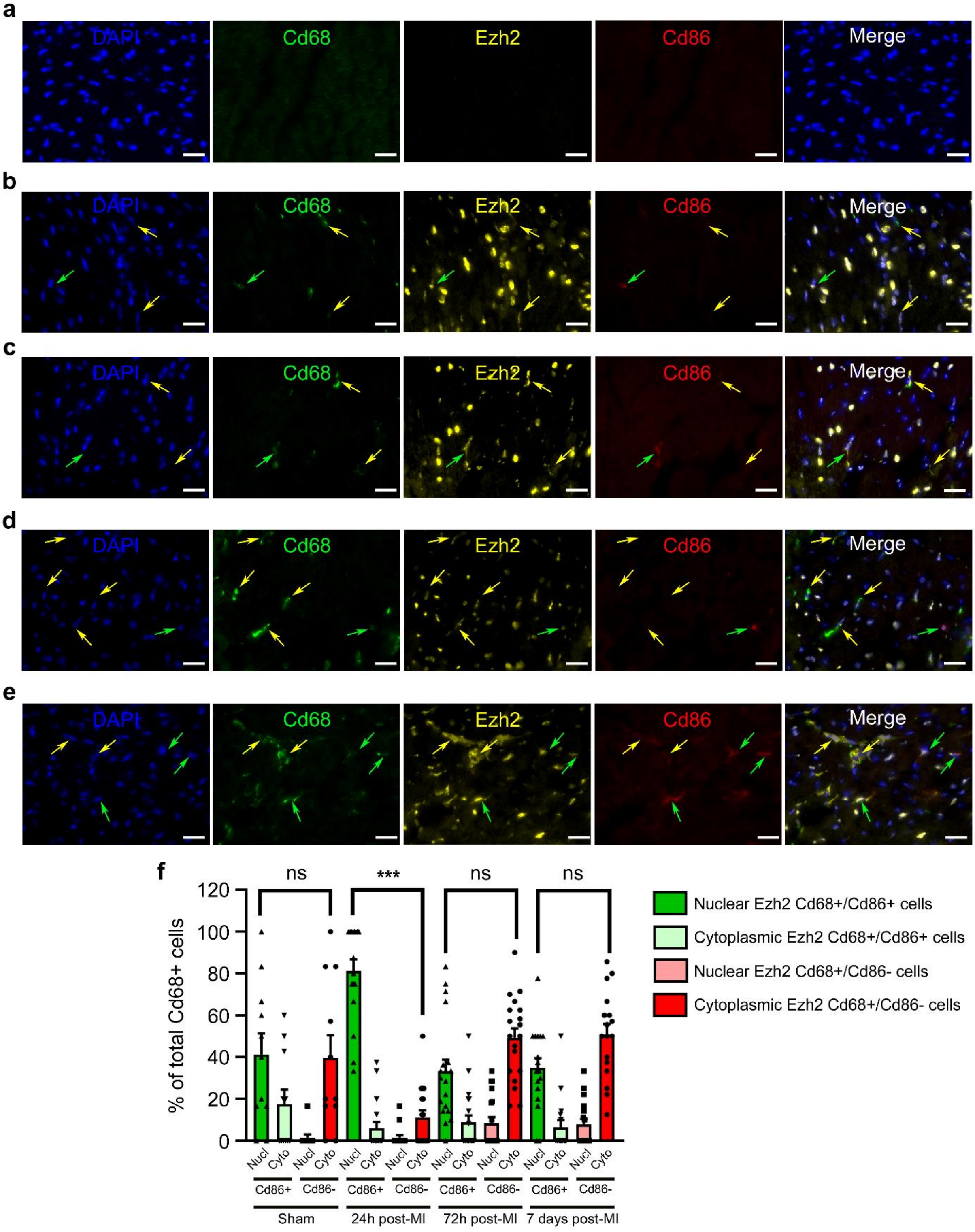

Figure S1: Ezh2 cellular localization in Cd68+/Cd86+ pro-inflammatory cardiac macrophages post-MI

Representative pictures of cardiac immunostaining for Ezh2 subcellular localization in $Cd68^+/Cd86^+$ pro-inflammatory macrophages in (a) negative auto-fluorescence control, (b) healthy sham mice, (c) 24h post-MI, (d) 72h post-MI and (e) 7 days post-MI. Nuclei were stained with DAPI (*blue*), macrophages were stained with Cd68 (*green*), macrophage subpopulation status was assessed by Cd86 (*red*) staining. Ezh2 (*yellow*) cellular localization was quantified (f) for either pro-inflammatory ($Cd68^+/Cd86^+$) or non-determined ($Cd68^+/Cd86^-$) macrophages at each time point. Arrows indicate pro-inflammatory macrophages (*green*) and non-determined (*yellow*), scale bars represent 25 µm. Data are represented as mean cell percentage of overall macrophages ($Cd68^+$) ± SEM of indicated random fields of view for each time point post-MI: Sham (n=11), 24h post-MI (n=17), 72h post-MI (n=19) and 7 days post-MI (n=17). Asterisk (*) indicates statistically significant difference between nuclear Ezh2 $Cd68^+/Cd86^+$ and cytoplasmic Ezh2 $Cd68^+/Cd86^-$ for each time point post-MI after Kruskal-Wallis test. The p values are depicted in the figure as follows: ***p <0.001; ns: non-significant. Source data are provided as a Source Data file.

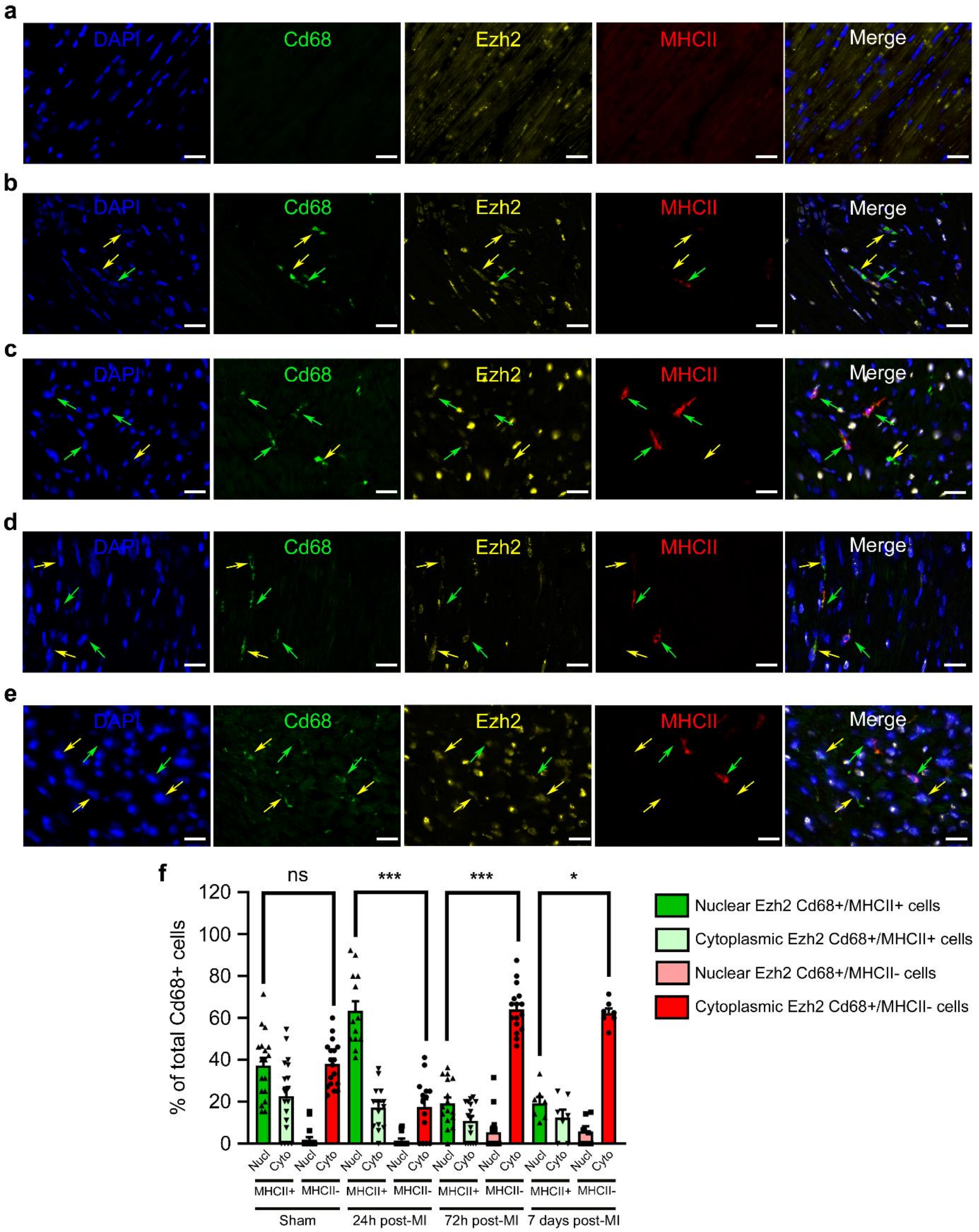

Figure S2: Cellular Ezh2 localization in Cd68+/MHCII+ pro-inflammatory cardiac macrophages post-MI

Representative pictures of cardiac immunostaining for Ezh2 subcellular localization in Cd68^{+}/MHCII^{+} pro-inflammatory macrophages in (a) negative auto-fluorescence

control, (b) healthy sham mice, (c) 24h post-MI, (d) 72h post-MI and (e) 7 days post-MI. Nuclei were stained with DAPI (*blue*), macrophages were stained with Cd68 (*green*), macrophage subpopulation status was assessed by MHCII (*red*) staining. Ezh2 (*yellow*) cellular localization was quantified (f) for either pro-inflammatory ($Cd68^+/MHCII^+$) or non-determined ($Cd68^+/MHCII^-$) macrophages at each time point. Arrows indicate pro-inflammatory macrophages (*green*) and non-determined (*yellow*), scale bars represent 25 µm. Data are represented as mean cell percentage of overall macrophages ($Cd68^+$) ± SEM of indicated random fields of view for each time point post-MI: Sham (n=18), 24h post-MI (n=14), 72h post-MI (n=16) and 7 days post-MI (n=7). Asterisk (*) indicates statistically significant difference between nuclear Ezh2 $Cd68^+/MHCII^+$ and cytoplasmic Ezh2 $Cd68^+/MHCII^-$ for each time point post-MI after Kruskal-Wallis test. The p values are depicted as asterisks in the figures as follows: ***p <0.001; *p <0.05; ns: non-significant. Source data are provided as a Source Data file.

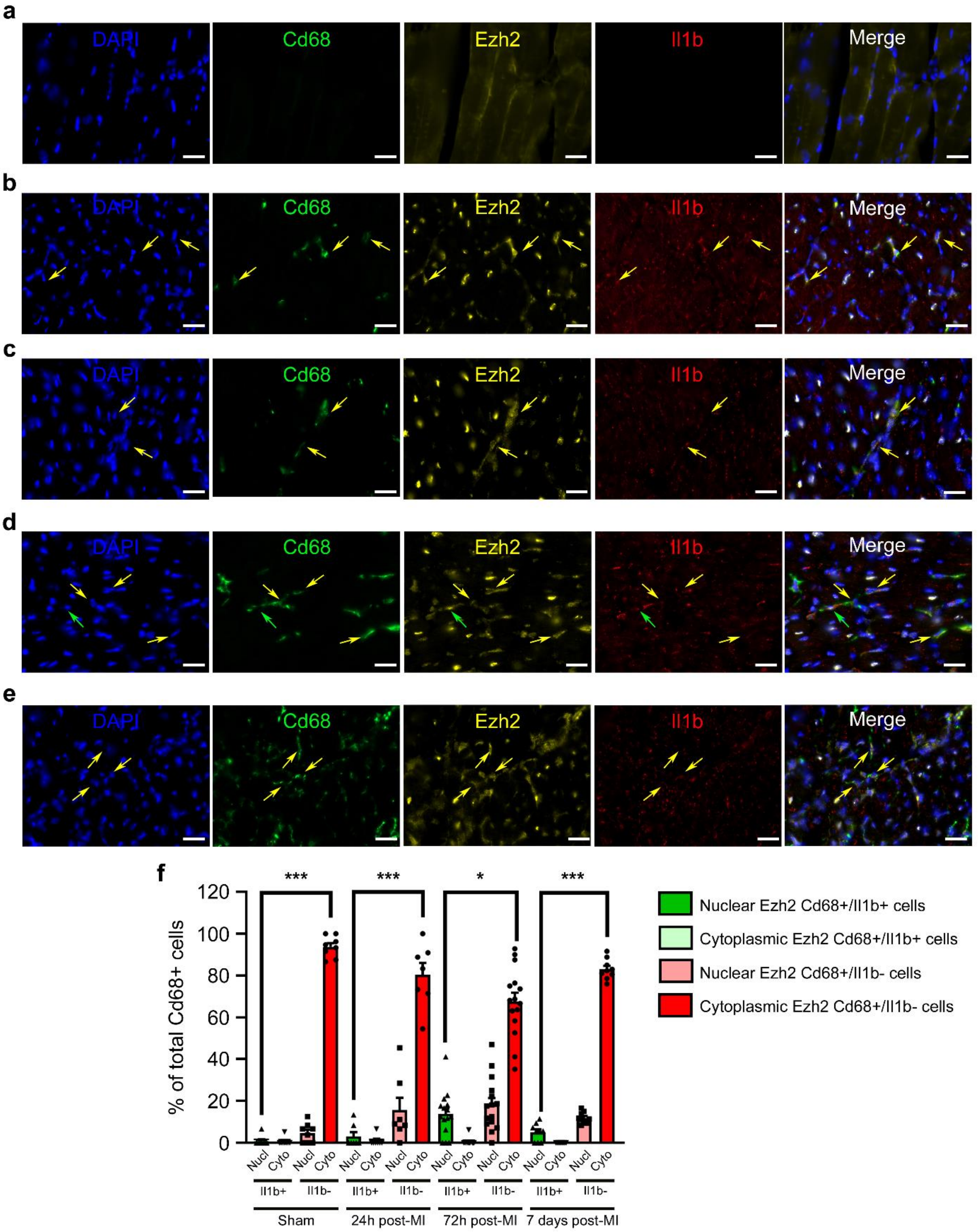

Figure S3: Cellular Ezh2 localization in Cd68+/Il1b+ pro-inflammatory cardiac macrophages post-MI

Representative pictures of cardiac immunostaining for Ezh2 subcellular localization in Cd68^{+}/Il1b^{+} pro-inflammatory macrophages in (a) negative auto-fluorescence control,

(b) healthy sham mice, (c) 24h post-MI, (d) 72h post-MI and (e) 7 days post-MI. Nuclei were stained with DAPI (*blue*), macrophages were stained with Cd68 (*green*), macrophage subpopulation status was assessed by Il1b (*red*) staining. Ezh2 (*yellow*) cellular localization was quantified (f) for either pro-inflammatory ($Cd68^{+}/Il1b^{+}$) or non-determined ($Cd68^{+}/Il1b^{-}$) macrophages at each time point. Arrows indicate pro-inflammatory macrophages (*green*) and non-determined (*yellow*), scale bars represent 25 µm. Data are represented as mean cell percentage of overall macrophages ($Cd68^{+}$) ± SEM of indicated random fields of view for each time point post-MI: Sham (n=8), 24h post-MI (n=7), 72h post-MI (n=15) and 7 days post-MI (n=9). Asterisk (*) indicates statistically significant difference between nuclear Ezh2 $Cd68^{+}/Il1b^{+}$ and cytoplasmic Ezh2 $Cd68^{+}/Il1b^{-}$ for each time point post-MI after Kruskal-Wallis test. The p values are depicted as asterisks in the figures as follows: ***p <0.001; *p <0.05; ns: non-significant. Source data are provided as a Source Data file.

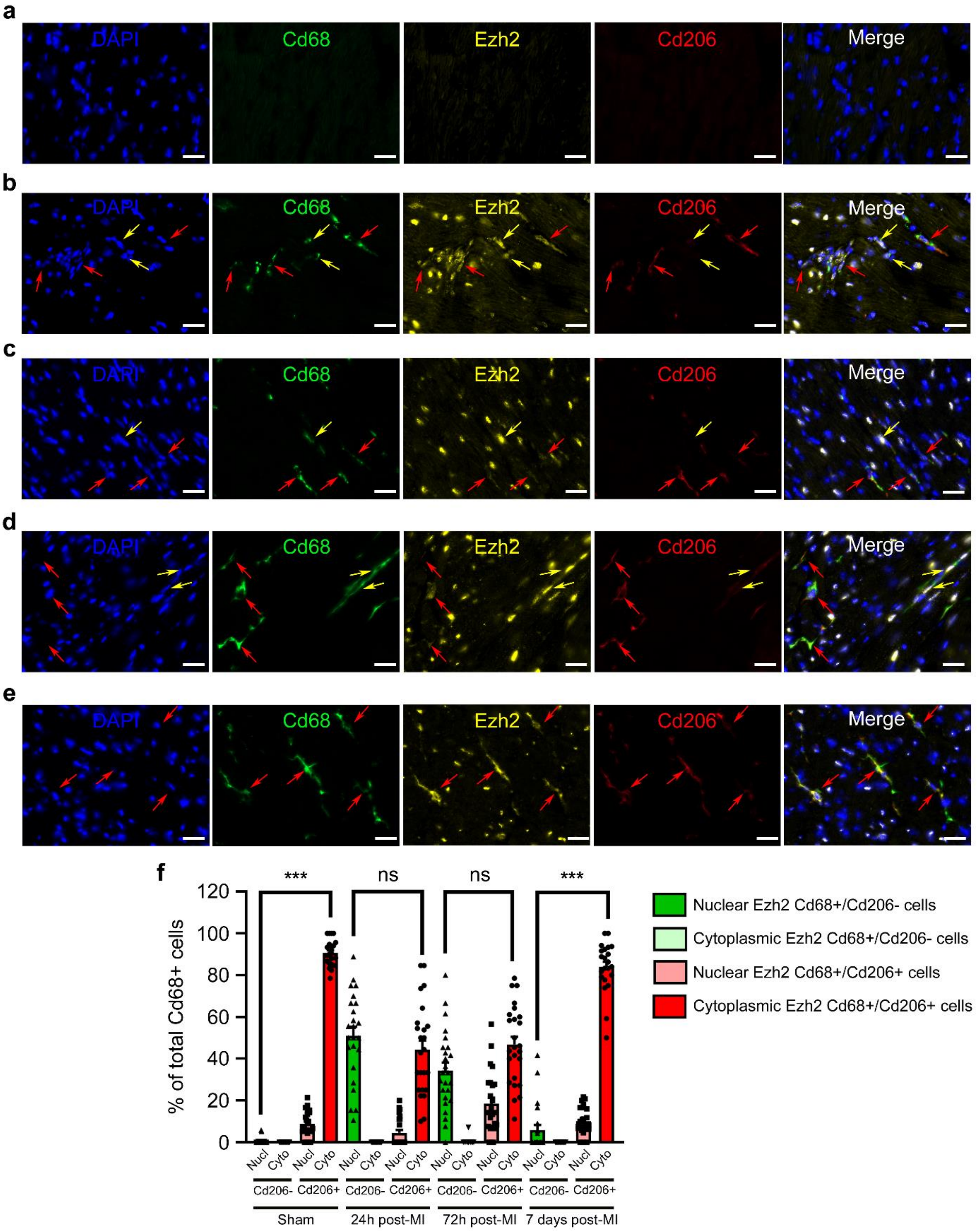

Figure S4: Cellular Ezh2 localization in Cd68+/Cd206+ immunomodulatory cardiac macrophages post-MI

Representative pictures of cardiac immunostaining for Ezh2 subcellular localization in Cd68^{+}/Cd206^{+} immunomodulatory macrophages in (a) negative auto-fluorescence

control, (b) healthy sham mice, (c) 24h post-MI, (d) 72h post-MI and (e) 7 days post-MI. Nuclei were stained with DAPI (*blue*), macrophages were stained with Cd68 (*green*), macrophage subpopulation status was assessed by Cd206 (*red*) staining. Ezh2 (*yellow*) cellular localization was quantified (f) for either immunomodulatory ($Cd68^+/Cd206^+$) or non-determined ($Cd68^+/Cd206^-$) macrophages at each time point. Arrows indicate immunomodulatory macrophages (*red*) and non-determined (*yellow*), scale bars represent 25 µm. Data are represented as mean cell percentage of overall macrophages ($Cd68^+$) ± SEM of indicated random fields of view for each time point post-MI: Sham (n=22), 24h post-MI (n=24), 72h post-MI (n=25) and 7 days post-MI (n=22). Asterisk (*) indicates statistically significant difference between nuclear Ezh2 $Cd68^+/Cd206^-$ and cytoplasmic Ezh2 $Cd68^+/Cd206^+$ for each time point post-MI after Kruskal-Wallis test. The p values are depicted as asterisks in the figures as follows: ***p <0.001; ns: non-significant. Source data are provided as a Source Data file.

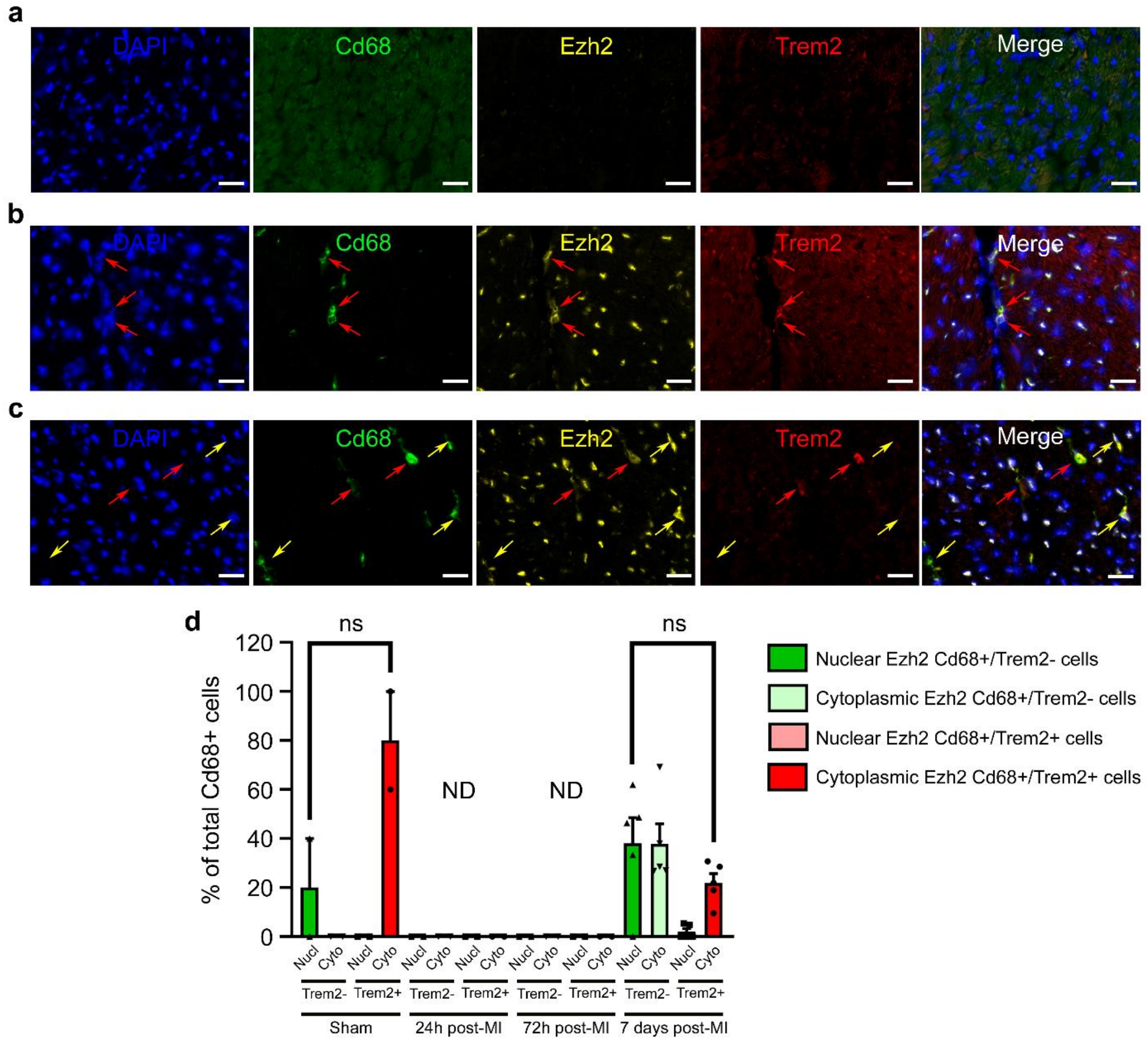

Figure S5: Cellular Ezh2 localization in Cd68+/Trem2+ immunomodulatory cardiac macrophages post-MI

Representative pictures of cardiac immunostaining for Ezh2 subcellular localization in $Cd68^{+}/Trem2^{+}$ immunomodulatory macrophages in (a) negative auto-fluorescence control, (b) healthy sham mice, (c) 7 days post-MI. Nuclei were stained with DAPI (*blue*), macrophages were stained with Cd68 (*green*), macrophage subpopulation status was assessed by Trem2 (*red*) staining. Ezh2 (*yellow*) cellular localization was quantified (d) for either immunomodulatory ($Cd68^{+}/Trem2^{+}$) or non-determined ($Cd68^{+}/Trem2^{-}$) macrophages at each time point. Arrows indicate immunomodulatory

macrophages (*red*) and non-determined (*yellow*), scale bars represent 25 μm. Data are represented as mean cell percentage of overall macrophages ($Cd68^+$) ± SEM of indicated random fields of view for each time point post-MI: Sham (n=2) and 7 days post-MI (n=5). Asterisk (*) indicates statistically significant difference between nuclear Ezh2 $Cd68^+$/$Trem2^-$ and cytoplasmic Ezh2 $Cd68^+$/$Trem2^+$ for each time point post-MI after Kruskal-Wallis test. The p values are depicted as asterisks in the figures as follows: ns: non-significant; ND: Not Determined. Source data are provided as a Source Data file.

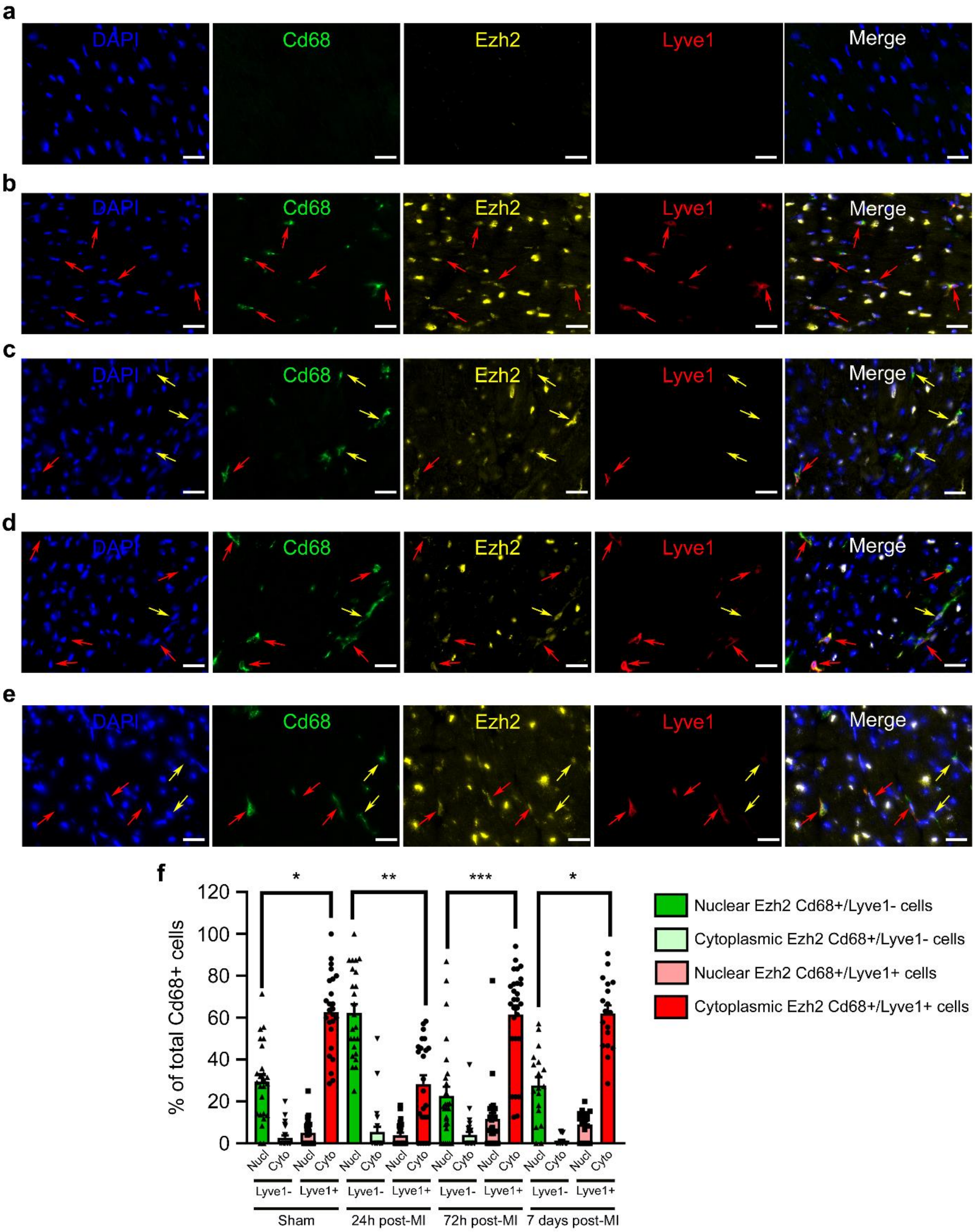

Figure S6: Cellular Ezh2 localization in Cd68+/Lyve1+ immunomodulatory cardiac macrophages post-MI

Representative pictures of cardiac immunostaining for Ezh2 subcellular localization in Cd68^{+}/Lyve1^{+} cardiac-resident macrophages in (a) negative auto-fluorescence

control, (b) healthy sham mice, (c) 24h post-MI, (d) 72h post-MI and (e) 7 days post-MI. Nuclei were stained with DAPI (*blue*), macrophages were stained with Cd68 (*green*), cardiac-resident macrophage subpopulation status was assessed by Lyve1 (*red*) staining. Ezh2 (*yellow*) cellular localization was quantified (f) for either resident ($Cd68^+/Lyve1^+$) or non-determined ($Cd68^+/Lyve1^-$) macrophages at each time point. Arrows indicate resident macrophages (*red*) and non-determined (*yellow*), scale bars represent 25 µm. Data are represented as mean cell percentage of overall macrophages ($Cd68^+$) ± SEM of indicated random fields of view for each time point post-MI: Sham (n=25), 24h post-MI (n=24), 72h post-MI (n=28) and 7 days post-MI (n=18). Asterisk (*) indicates statistically significant difference between nuclear Ezh2 $Cd68^+/Lyve1^-$ and cytoplasmic Ezh2 $Cd68^+/Lyve1^+$ for each time point post-MI after Kruskal-Wallis test. The p values are depicted as asterisks in the figures as follows: ***p <0.001; **p <0.01; *p <0.05. Source data are provided as a Source Data file.

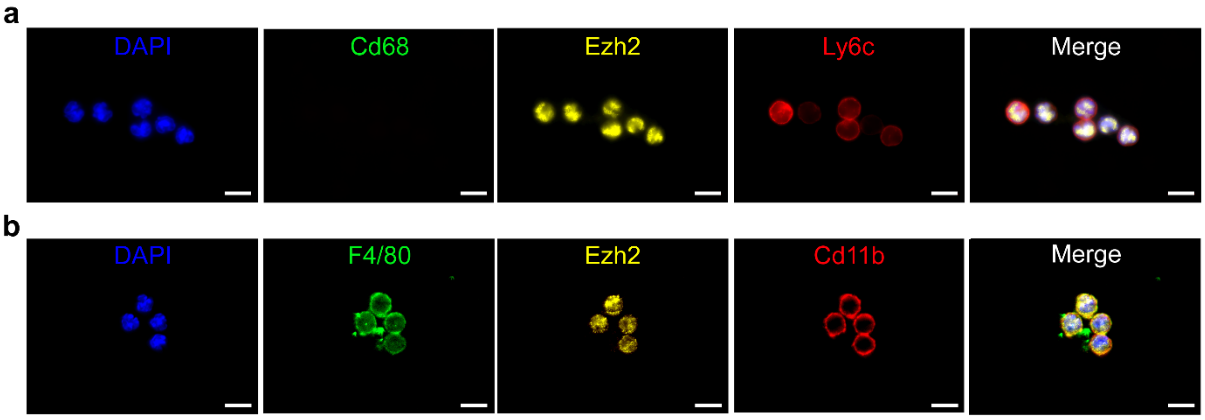

Figure S7: Cellular Ezh2 localization in mouse selected non-adherent and adherent monocytes *in vitro*

Representative pictures of immunostaining for Ezh2 in (a) non-adherent, (b) adherent monocytes *in vitro*. Nuclei were stained with DAPI (*blue*), monocytes were identified based on Ly6c or Cd11b (*red*, panels a and b) combined either with Cd68 (green, panels a) or F4/80 (*green*, panel b) expression, and Ezh2 (yellow) cellular localization was examined in both types of monocytes. Scale bars represent 10 µm. These immunostainings have been reproduced at least 3 times from different mice.

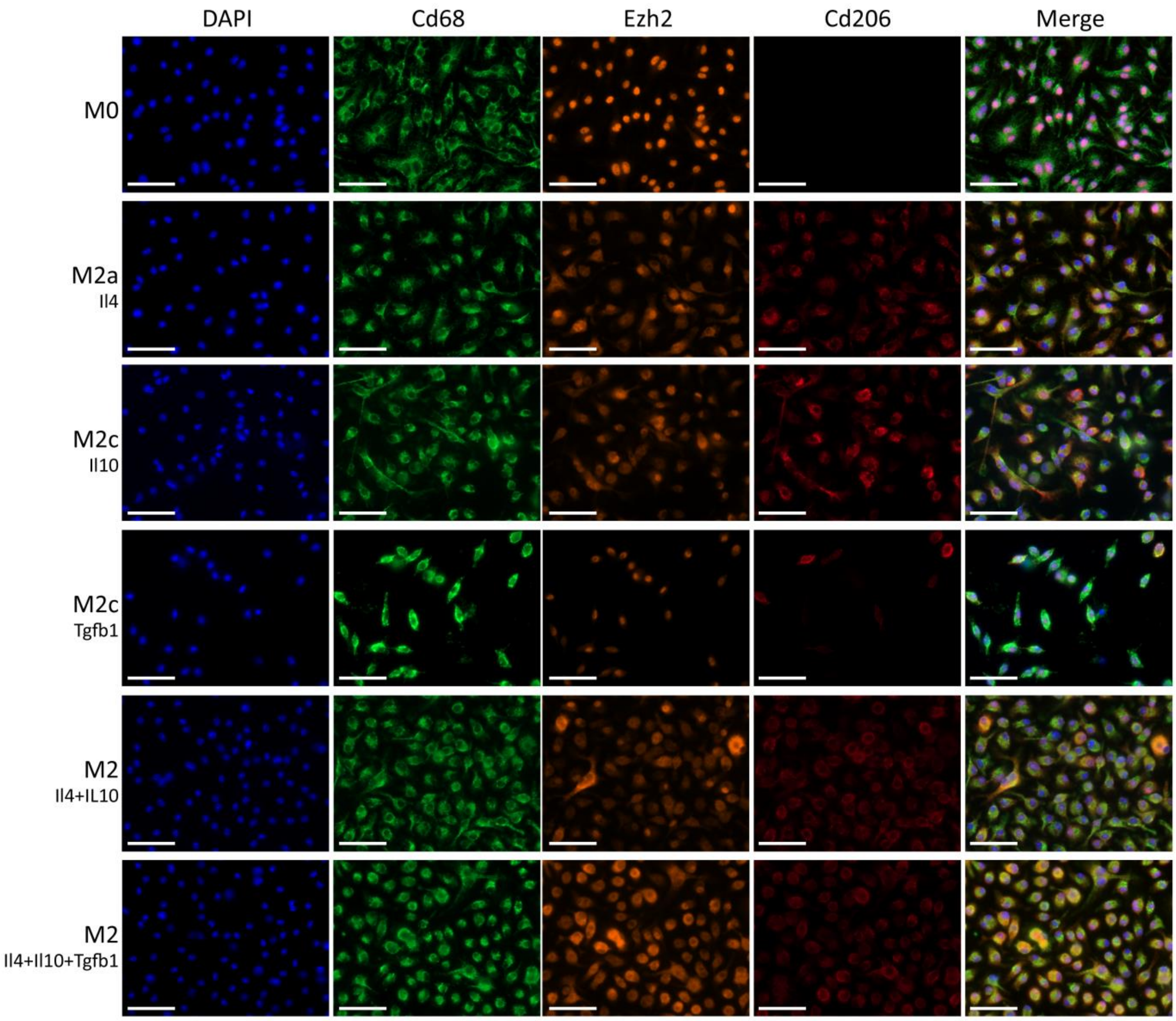

Figure S8: Ezh2 cytoplasmic translocation is not restricted to a single M2 macrophage phenotype

Immunostaining representative pictures of mouse bone marrow-derived *in vitro* matured non-polarized M0, M2a (Il4), M2c (Il10 or Tgfb1) and M2 differentially (Il4 and Il10 with or without Tgfb1) polarized macrophages. Nuclei were stained with DAPI (blue), macrophages with Cd68 (*green*) and M2 polarization was assessed based on Cd206 (*red*) expression. Ezh2 (orange) cellular localization was observed in all cell type but only appeared in the cytoplasm of M2 macrophages. Scale bars represent 50 µm. These immunostainings have been reproduced at least 3 times from different mice with similar results.

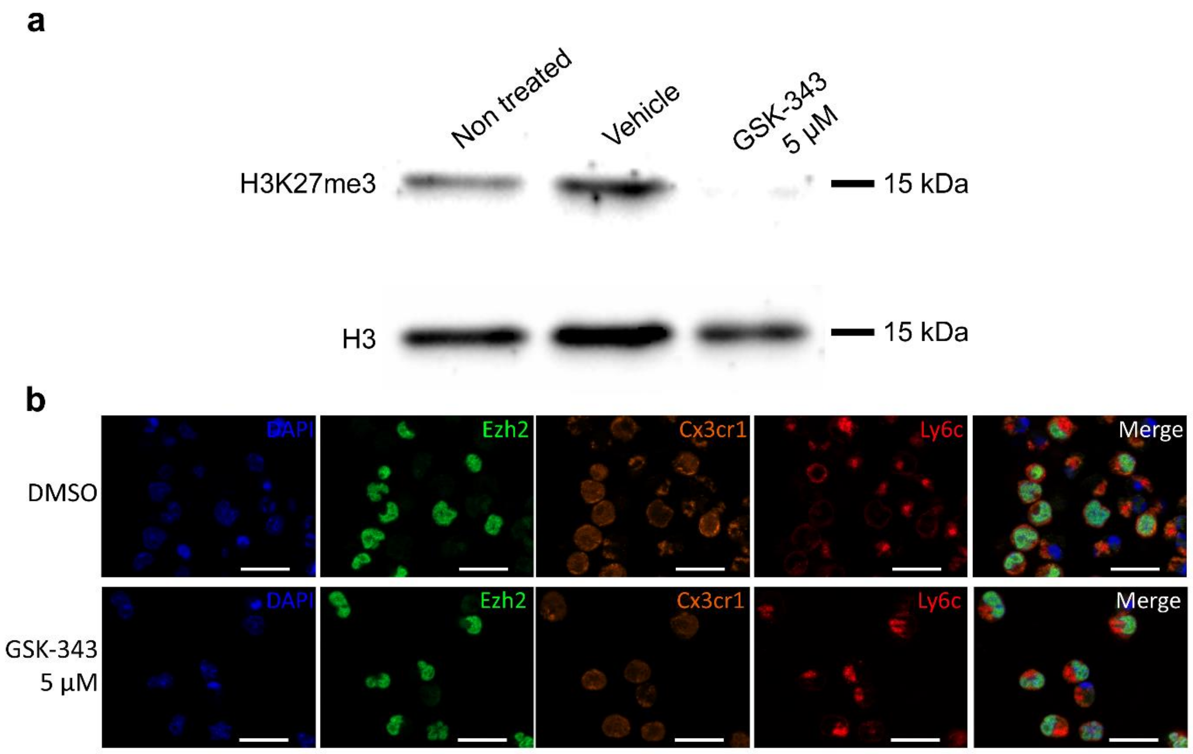

Figure S9: GSK-343 reduces H3K27me3 levels without altering Ezh2 expression in murine monocytes

(a) Mouse monocytes (TIB-204 cell line) treatment with GSK-343 in decreases histone H3K27me3 levels, as determined by western blot. Molecular weights are indicated in kDa. Total H3 histone serves as loading control. This experiment has been performed only once. (b) Representative pictures of immunostaining of mouse monocytes treated for 72h with vehicle or GSK-343 (5 µM). Nuclei were stained with DAPI (*blue*), Ezh2 (*green*) cellular localization was analyzed in Cx3cr1 (*orange*) versus Ly6c (*red*) expressing monocyte subsets. Scale bars represent 25 µm. These immunostainings have been reproduced at least 3 times independently with similar results. Source data are provided as a Source Data file.

	Non-CAD Patient#1	Non-CAD Patient#2	Non-CAD Patient#3	Overall n = 3
Erythrocytes (10^{12}¢/l)	3.98	4.22	5.01	4.40±0.54
Total leucocytes (10^{9}¢/l)	5.4	4.9	6.3	5.53±0.71
Neutrophils (10^{9}¢/l)	3.89	3.13	4.53	3.85±0.70
Eosinophils (10^{9}¢/l)	0.12	0.01	0.12	0.08±0.06
Basophils (10^{8}¢/l)	0.03	0	0.06	0.03±0.03
Lymphocytes (10^{9}¢/l)	0.65	0.98	1.1	0.91±0.23
Monocytes (10^{9}¢/l)	0.71	0.78	0.49	0.66±0.15
Platelets (10^{9}¢/l)	276	137	209	207.33±69.51

Supplementary table 1: non-CAD patient characteristics used for RNA-seq analysis

The gender (male/female) ratio was 1/2 with a median age of 65 years old.

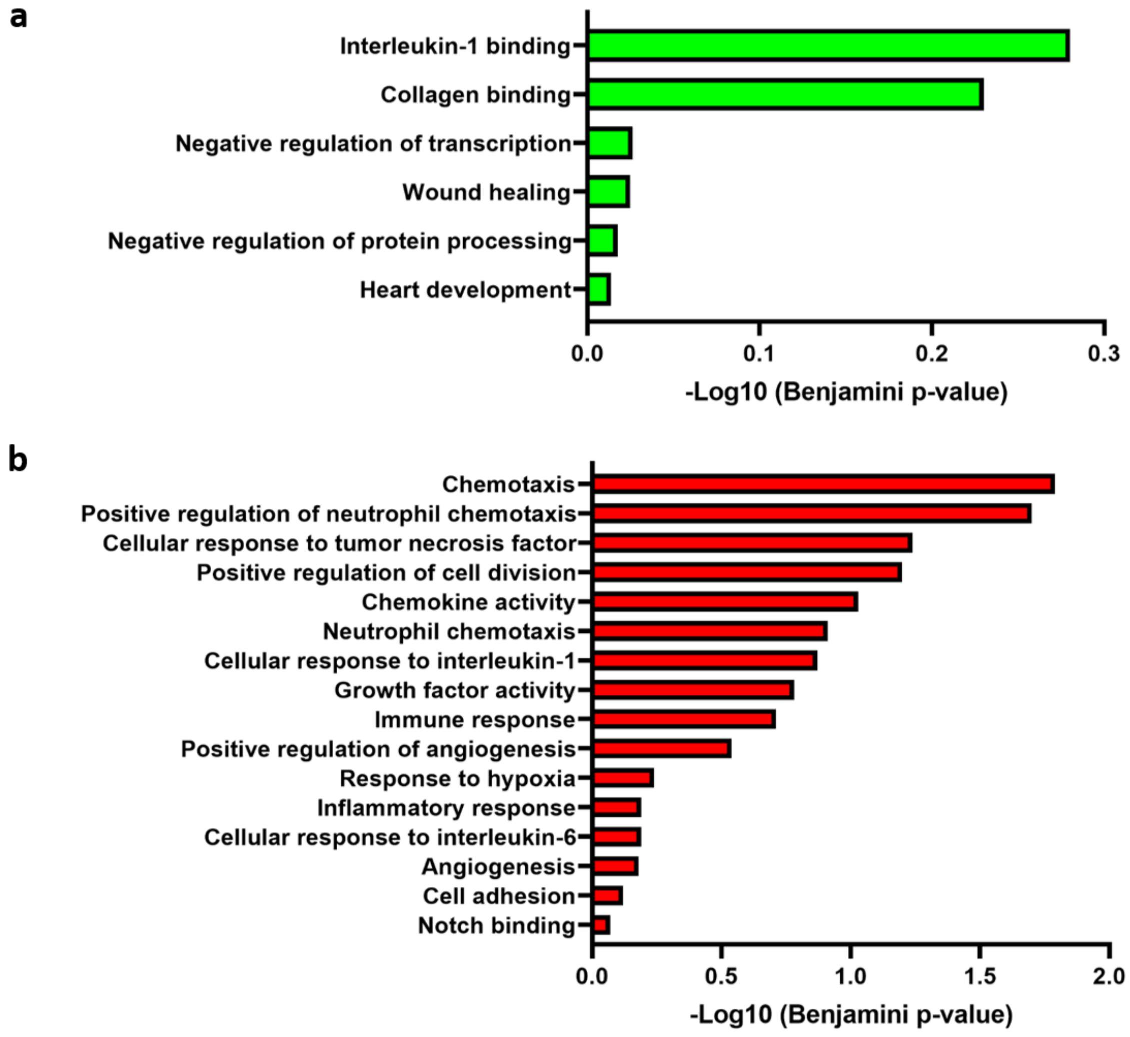

Figure S10: GSK-343 treatment of human monocytes promotes expression of genes regulating chemotaxis and angiogenesis

Representative Gene Ontology (GO) Biological Process categories significantly enriched for down- (a) and up-regulated (b) genes in human monocytes treated with GSK-343 as determined by mRNA-Seq. The bar graph represents the -log10 (Benjamini-Hochberg adjusted p-value), obtained from DAVID gene-enrichment in functional annotation terms after Fisher's Exact test.

	Group 1 Non-CAD n = 20	Group 2 CAD n = 21	Group 3 AMI n = 7	Overall n = 48
Erythrocytes (10^{12}¢/l)	4.46±0.50	4.50±0.54	5.31±0.61	4.59±0.61
Total leucocytes (10^{9}¢/l)	6.68±1.82	7.75±1.79	13.22±6.26	8.10±3.54
Neutrophils (10^{9}¢/l)	4.22±1.56	5.29±1.52	10.08±6.03	5.55±3.26
Eosinophils (10^{9}¢/l)	0.19±0.15	0.20±0.13	0.07±0.06	0.18±0.14
Basophils (10^{8}¢/l)	0.45±0.24	0.51±0.31	0.46±0.39	0.47±0.29
Lymphocytes (10^{9}¢/l)	1.59±0.70	1.62±0.57	2.27±1.66	1.70±0.86
Monocytes (10^{9}¢/l)	0.63±0.18	0.69±0.25	0.76±0.30	0.68±0.23
Platelets (10^{9}¢/l)	234.9±58.9	227.4±51.2	279.0±94.1	238.1±63.0

Supplementary table 2: patient characteristics

The overall gender (male/female) ratio was 31/17 with a median age of 79 ranging from 57 to 94 years old.

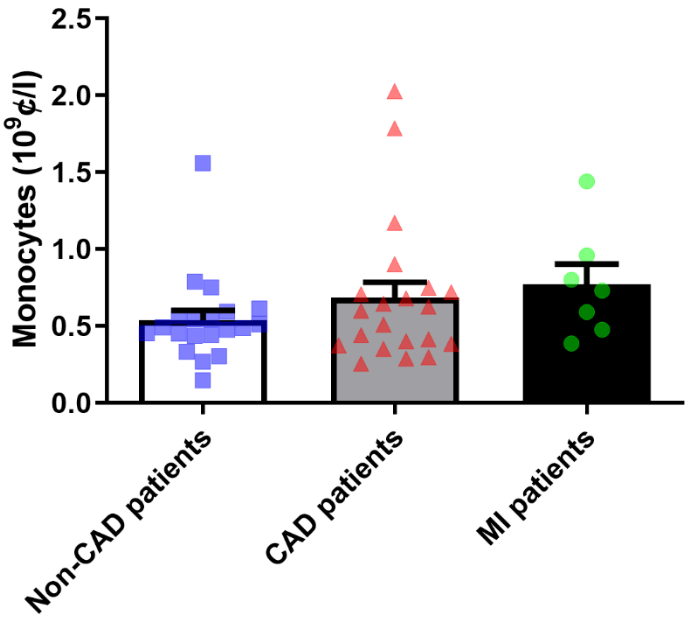

Figure S11: Levels of selected circulating monocytes in MI patients as compared to non-CAD and CAD patients

Quantification of blood-derived human monocytes, obtained after negative magnetic selection. Data is represented as mean number of live selected monocytes obtained per ml of collected blood, Non-CAD (n=20), CAD (n=21) and MI (n=7) patients ± SEM. Source data are provided as a Source Data file.

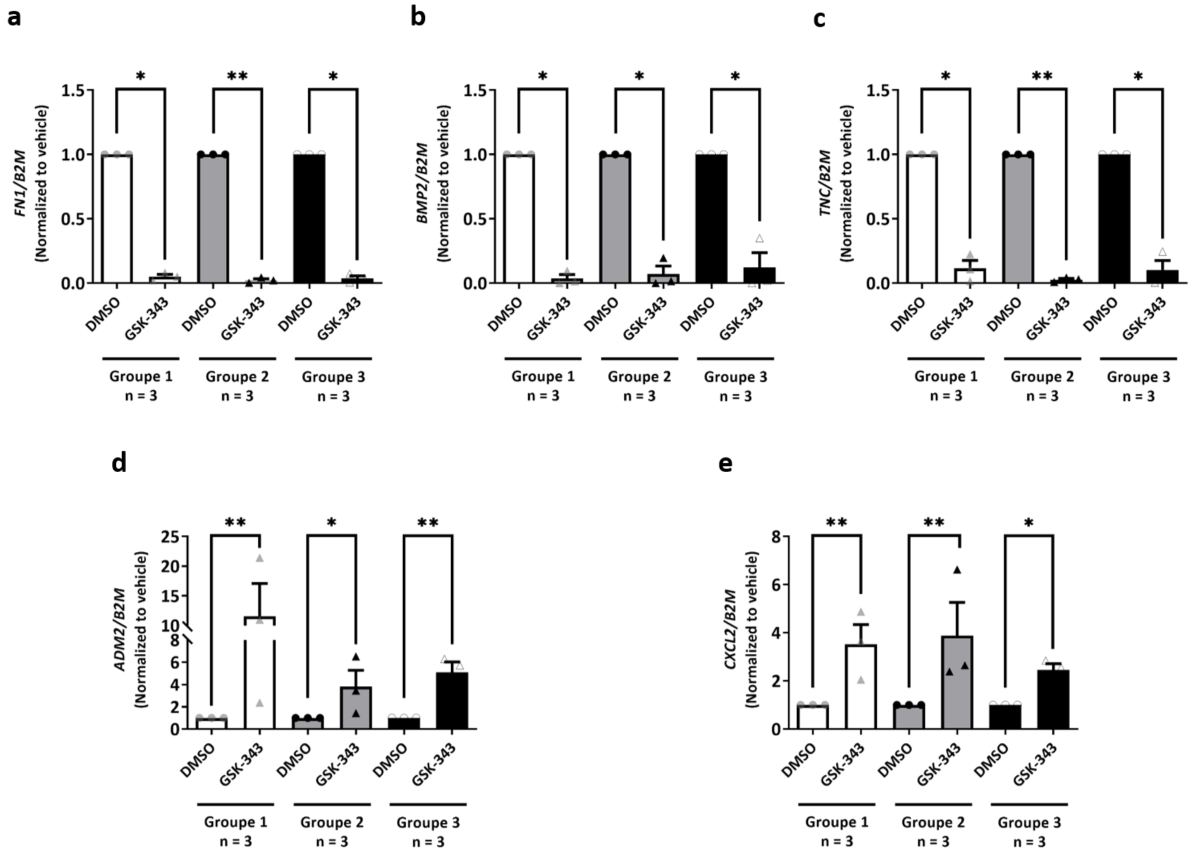

Figure S12: Altered expression of genes identified by RNA-seq upon GSK-343 treatment of human monocytes

RT-qPCR measured expression levels of *FN1* (a), *BMP2* (b), *TNC* (c), *ADM2* (d) and *CXCL12* (e) in human monocytes from different patient groups (Group 1: Non-CAD (white), Group 2: CAD (grey) and Group 3: AMI (black)) treated *in vitro* with vehicle or GSK-343. Data are presented as mean expression of vehicle treated monocytes ± SEM, with *B2M* serving as internal control of four independent experiments corresponding to three different donors per group performed in duplicate (n=3). For all panels, the p values determined by Kruskal-Wallis test are depicted as asterisks as follows: **p <0.01; *p <0.05. Source data are provided as a Source Data file.

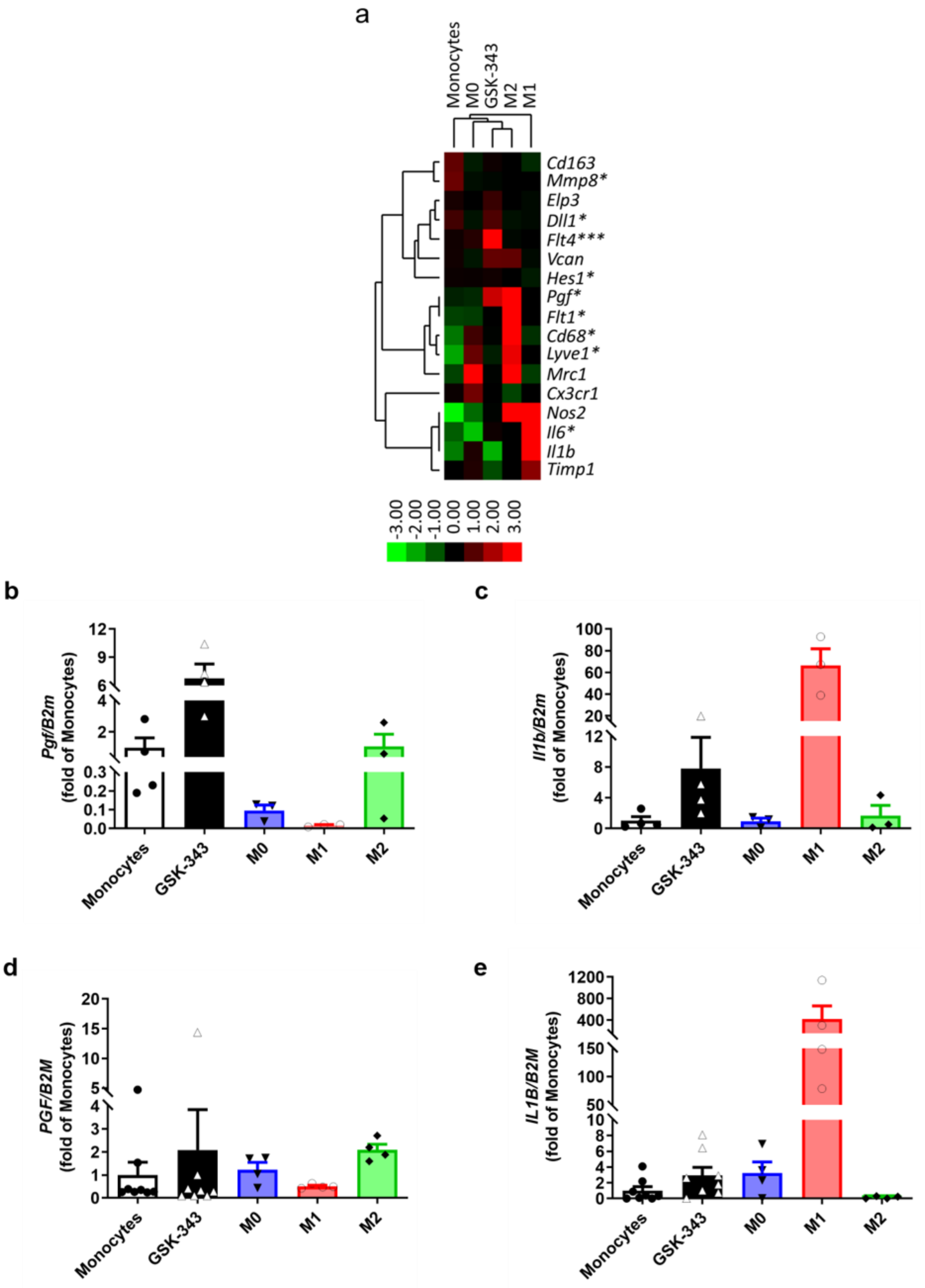

Figure S13: EZH2 inhibition with GSK-343 brings monocyte gene expression profile closer to M2 identity

Gene expression in monocytes treated with vehicle or GSK-343 or following *in vitro* differentiation of monocytes into M0, M1 or M2 macrophages from TIB-204 monocyte mouse cell line (a) or mouse primary circulating selected monocytes (b and c) or human primary circulating selected monocytes (d and e) was measured by RT-qPCR. Data are presented as a targeted transcriptomic array of selected genes for each cell type categories derived from TIB-204 (a, n=4) or mean expression reported to vehicle treated monocytes ± SEM for mouse primary circulating selected monocytes (b and c, n=4 for monocytes and GSK-343 conditions, n=3 for M0, M1 and M2 conditions) or human primary circulating selected monocytes (d and e, n=8 for monocytes and GSK-343 conditions, n=4 for M0, M1 and M2 conditions). Asterisk (*) symbols indicates statistically significant difference compared to monocytes condition after Kruskal-Wallis test as follows: ***p <0.001; **p <0.01; *p <0.05; ns: non-significant. Source data are provided as a Source Data file.

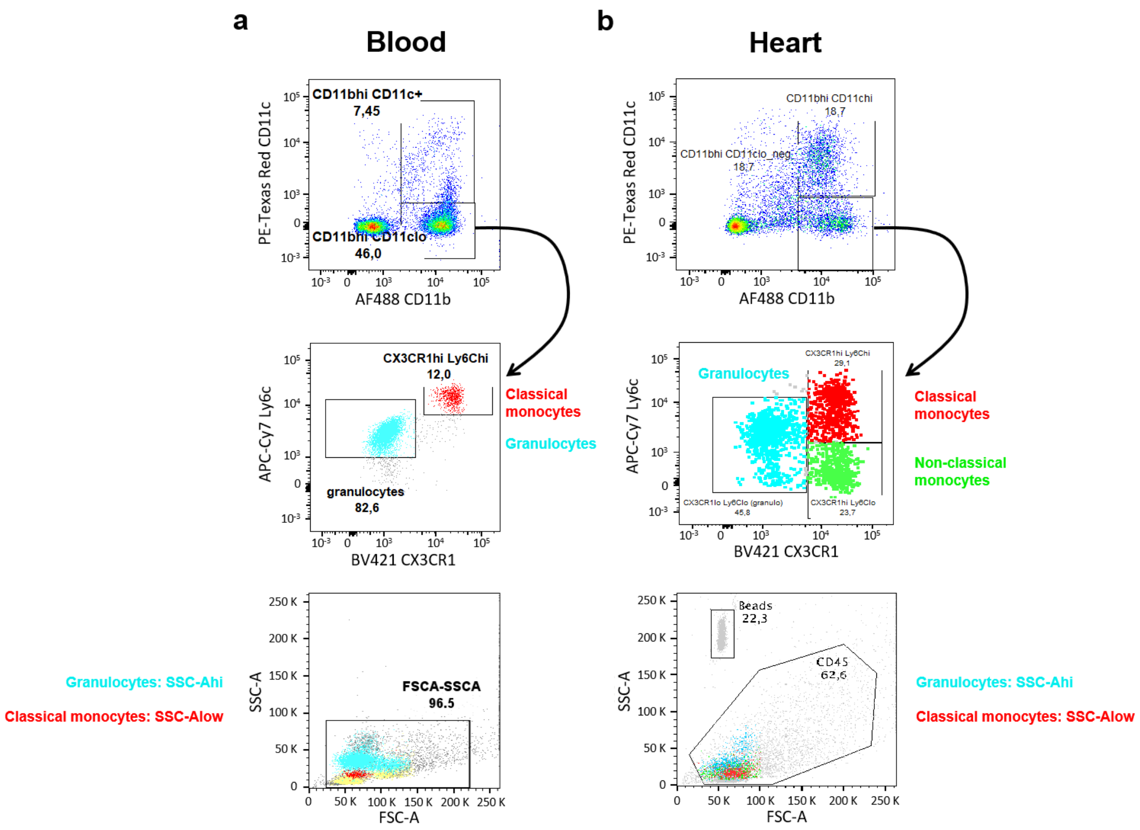

Figure S14: Flow cytometry gating strategy for mouse peripheral blood samples and heart samples

Flow cytometry gating strategy for myeloid cell quantification from peripheral blood (a) and heart tissue (b) samples. After peripheral blood ammonium-chloride-potassium red blood cell lysis and heart enzymatic dissociation, cells blocked stained with appropriate antibodies as described in the methods section. Classical/inflammatory and non-classical monocytes/macrophages were defined as $CD45^+/CD3^-/CD19^-/CD11b^+/CD11c^-/Ly6C^{hi}/Cx3cr1^{hi}$ and $CD45^+/CD3^-/CD19^-/CD11b^+/CD11c^-/Ly6C^{lo}/Cx3cr1^{hi}$, respectively as described in the methods section.

	Sham (n=4)	3 days post-MI		8 days post MI	
		Vehicle (n=7)	GSK-343 (n=5)	Vehicle (n=5)	GSK-343 (n=8)
**Cardiomyocyte sizes (µm²)**	398.5 ± 19.5	450.86 ± 28.9 ns	492.50 ± 12.8 ns	546.80 ± 43.80 *	441.87 ± 23.6 #
**Capillary density (Blood vessels/mm²)**	2439.5 ± 179.6	1681.28 ± 179.3 ***	1783.40 ± 228.6 ns	1521.0 ± 189.0 ***	1774.25 ± 199.85 ns
**Open lymphatic vessel density (vessels/mm²)**	5.47 ± 1.25	7.17 ± 2.09 ns	5.87 ± 2.02 ns	10.34 ± 2.33 p=0.07	6.68 ± 1.97 ns
**Total macrophages (cells/field)**	23.48 ± 1.77	30.58 ± 1.88 *	38.22 ± 3.26 p=0.11	38.98 ± 4.16 ***	39.76 ± 5.05 ns

20. Supplementary table 3: Histological cardiac parameters at 7 days post-MI in mice treated or not with GSK-343

Quantitative analysis of histologic cardiac parameters in border zone (BZ) of sham, vehicle and GSK-343 treated mice at 3- and 8-days post-MI. Data are presented as means ± SEM. Asterisk (*) and hashtag (#) symbols indicates statistically significant difference compared to sham and vehicle condition respectively after Kruskal-Wallis test. # p <0.05, ns non-significant, * p <0.05*** p <0.001.

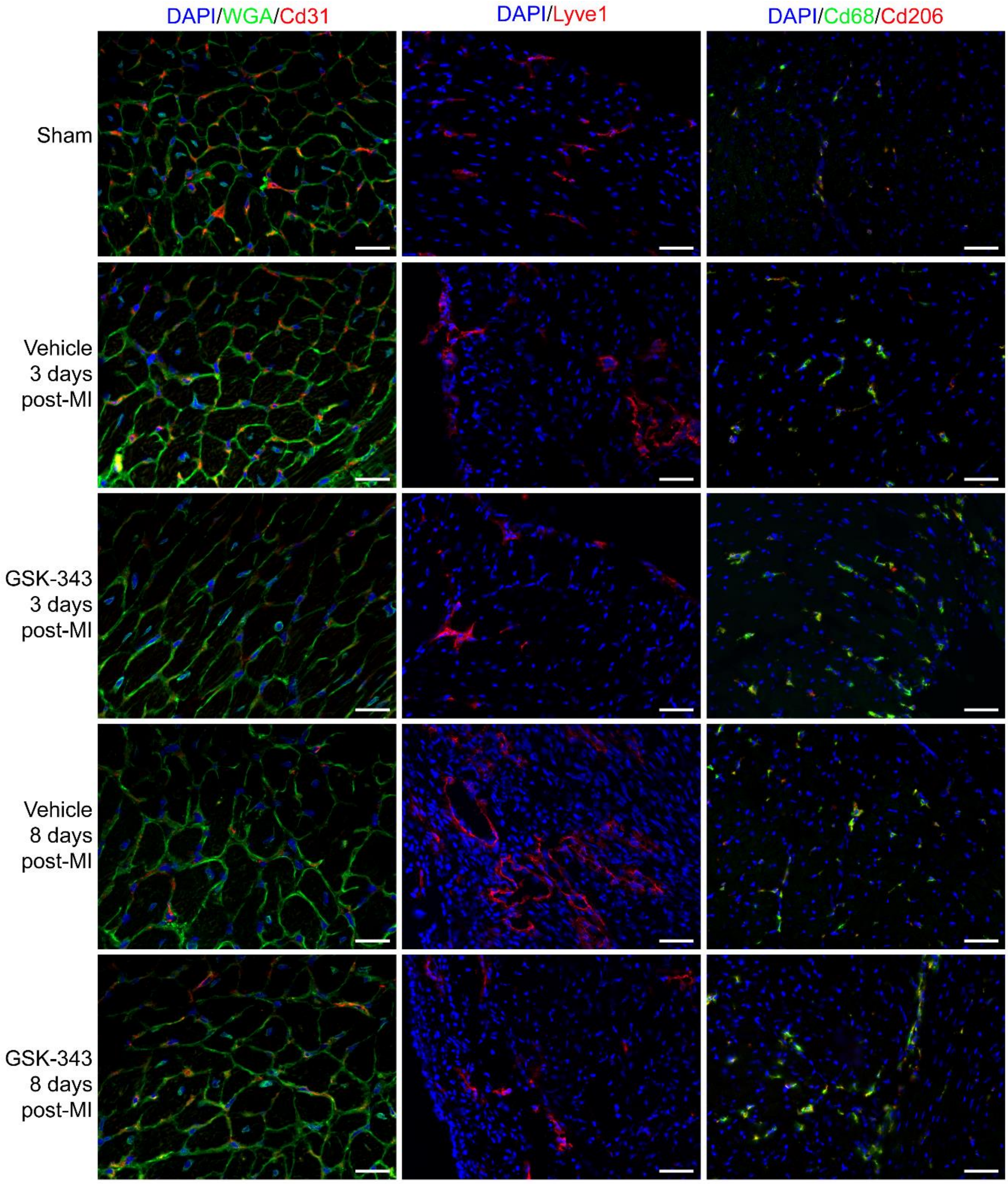

Figure S15: Examples of immunohistochemical analyses in mouse hearts at 7 days post-MI

Representative pictures of cardiac immunostaining for cardiomyocyte sizes and vascular capillary density (left panel), open lymphatic vessel density (center panel) and macrophage density (right panel) in sham mice and either 3 or 8 days post-MI

following treatment with GSK-343 or its vehicle. For all panels nuclei were stained with DAPI (*blue*), on left panel cardiomyocytes were stained with WGA (*green*) and capillaries were observed with Cd31 (*red*), on the center panel lymphatic vessels were visualized with Lyve1 (*red*) and finally on the right panel macrophages were stained with Cd68 (*green*) with immunomodulatory subset visualized using Cd206 (*red*), scale bars represent 100 μm. These pictures are representative of several different mice: sham (n=4), vehicle 3 days post-MI (n=7), GSK-343 3 days post-MI (n=5), vehicle 8 days post-MI (n=5), GSK-343 8 days post-MI (n=8) as shown by the quantifications in supplementary data 6.

Gene function	Gene name	3 days post-MI			8 days post MI		
		Sham	Vehicle	GSK-343	Sham	Vehicle	GSK-343
Inflammation	*Ccl2*	1.00 ± 0.19	4.41 ± 0.47 ***	4.08 ± 0.37 ***	See figure 6b		
	Ccl21	1.00 ± 0.23	0.65 ± 0.04 ns	0.62 ± 0.07 ns	See figure 6b		
	Il1b	1.00 ± 0.19	1.66 ± 0.14 **	1.51 ± 0.10 ns	See figure 6b		
	Il6	1.00 ± 0.28	3.97 ± 0.32 ***	5.31 ± 0.58 ***	See figure 6b		
	Il10	1.00 ± 0.21	1.62 ± 0.14 *	1.67 ± 0.14 *	1.00 ± 0.13	1.09 ± 0.11 ns	0.97 ± 0.10 ns
	Irf4	1.00 ± 0.25	0.46 ± 0.03 ***	0.51 ± 0.04 ***	1.00 ± 0.10	0.58 ± 0.03 ***	0.66 ± 0.04 **
	Tnf	1.00 ± 0.27	1.50 ± 0.10 ns	1.47 ± 0.09 ns	1.00 ± 0.11	1.56 ± 0.12 *	1.52 ± 0.13 ns
Angiogenesis	*Dll1*	1.00 ± 0.30	0.59 ± 0.03 ns	0.61 ± 0.04 ns	See figure 6b		
	Vegfa	1.00 ± 0.23	0.65 ± 0.04 **	0.74 ± 0.06 ns	1.00 ± 0.08	0.69 ± 0.03 *	0.71 ± 0.05 *
Fibrosis	*Fn1*	1.00 ± 0.31	5.64 ± 0.65 ***	6.66 ± 0.81 ***	See figure 6b		
	TnC	1.00 ± 0.29	10.63 ± 1.91 ***	10.92 ± 2.15 ***	See figure 6b		
Cardiac dysfunction	*Mb*	1.00 ± 0.19	0.61 ± 0.03 ns	0.63 ± 0.05 ns	See figure 6b		
	Nppa	1.00 ± 0.29	2.04 ± 0.26 ns	2.01 ± 0.25 ns	1.00 ± 0.30	2.59 ± 0.38 **	2.19 ± 0.29 ns
	Nppb	1.00 ± 0.18	1.77 ± 0.19 ns	2.18 ± 0.27 *	See figure 6b		
	Tnni3	1.00 ± 0.20	0.61 ± 0.03 ***	0.63 ± 0.06 **	See figure 6b		
	Tnnt2	1.00 ± 0.28	0.96 ± 0.06 ns	1.07 ± 0.17 ns	1.00 ± 0.10	0.66 ± 0.03 ns	0.77 ± 0.06 ns

Supplementary table 4: Altered gene expression of pathways related to Inflammation, angiogenesis, fibrosis and cardiac dysfunction in mice at 7 days post-MI

Cardiac transcript levels of indicated genes measured by RT-qPCR from sham (n=8), or vehicle (n=16) or GSK-343-treated (n=18) mice at 3 and 8 days after MI. Values expressed as mean percentages of sham ± SEM, with *B2m* serving as internal control. Asterisk (*) symbols indicates statistically significant difference compared to sham condition after Kruskal-Wallis test. ns non-significant, * p <0.05, *** p <0.001.

	3 days post-MI			7 days post MI		
	**Sham**	**Vehicle**	**GSK-343**	**Sham**	**Vehicle**	**GSK-343**
**LV dilatation index**	0.190 ± 0.01	0.166 ± 0.06 ns	0.180 ± 0.04 ns	See figure 6e		
**LVED (mm)**	3.75 ± 0.09	4.27 ± 0.10 *	4.21 ± 0.09 ns	See figure 6f		
**LVES (mm)**	2.94 ±0.10	3.66 ± 0.12 **	3.49 ± 0.11 ns	See figure 6g		
**LVFS (%)**	21.49 ± 1.73	14.50 ± 1.43 *	15.94 ± 1.43 ns	See figure 6h		
**LVEF (%)**	44.09 ± 3.10	30.19 ± 2.76 *	33.39 ± 2.70 ns	See figure 6i		
**Cardiac output (ml/min)**	9.45 ± 1.32	9.98 ± 0.94 ns	10.59 ± 0.90 ns	13.33 ± 1.05	11.07 ± 1.31 ns	12.04 ± 1.29 ns
**Heart rate (bpm)**	378.7 ± 18.57	394.8 ± 11.66 ns	406.0 ± 7.41 ns	410.4 ± 12.34	425.2 ± 14.22 ns	429.6 ± 13.24 ns
**LV mass (mg)**	72.21 ± 1.61	85.32 ± 5.51 ns	94.65 ± 2.88 ns	74.98 ± 6.51	108.10 ± 5.34 *	100.80 ± 7.38 ns
**Stroke volume (µl)**	26.54 ± 2.47	24.82 ± 2.36 ns	26.07 ± 2.05 ns	32.18 ± 1.97	26.92 ± 3.18 ns	27.91 ± 2.54 ns

23. Supplementary table 5: Echocardiographic cardiac evaluation at 3 and 7 days post-MI in mice

Quantitative analysis of echocardiographic cardiac parameters in sham (n=8), vehicle (n=14) and GSK-343 (n=18) treated mice at 3- and 7-days post-MI. Data are presented as means ± SEM. Asterisk (*) symbols indicates statistically significant

difference compared to sham condition after Kruskal-Wallis test. ns non-significant, * $p < 0.05$, *** $p < 0.001$.

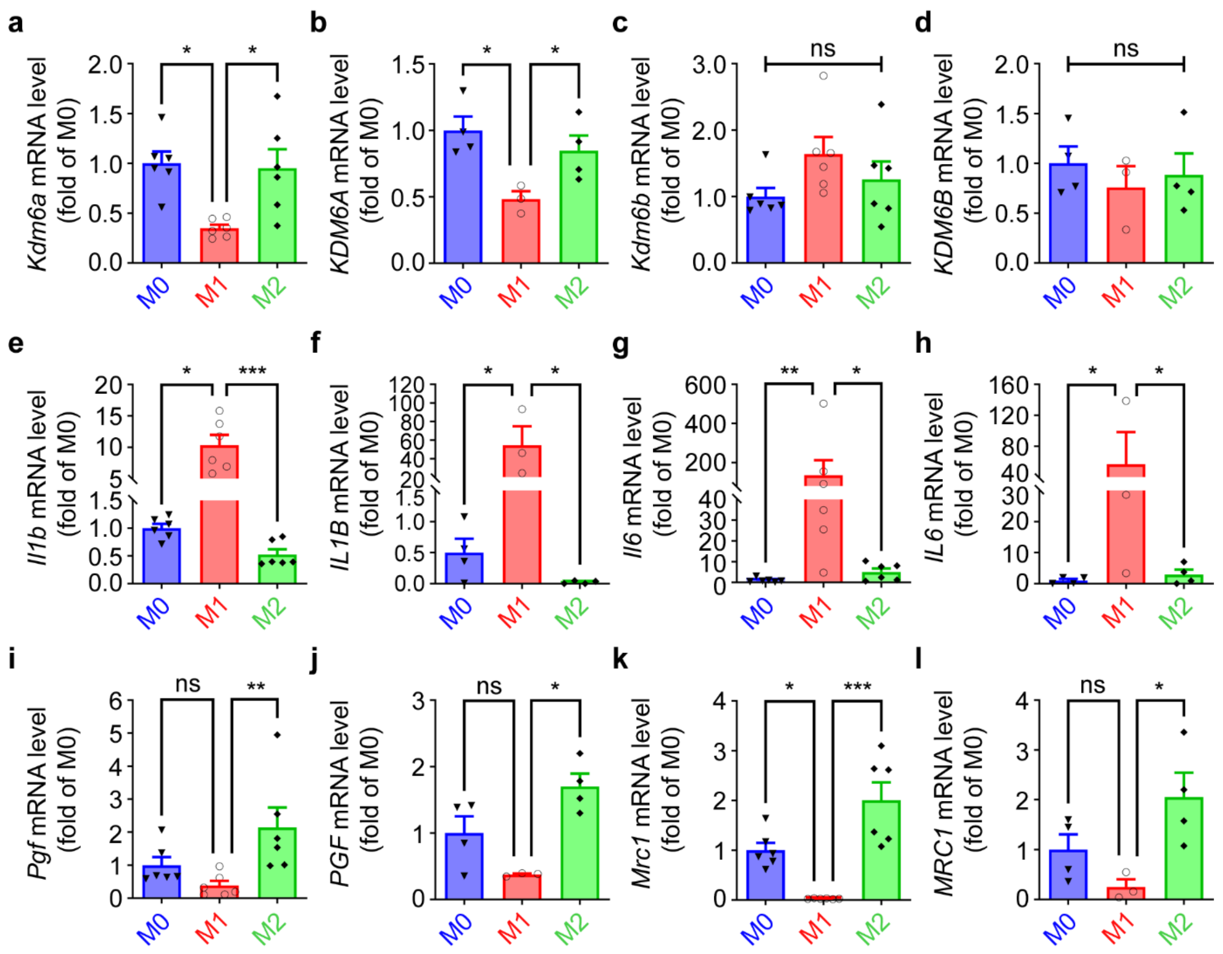

Figure S16: Gene expression analysis of UTX (Kdm6a), JMJD3 (Kdm6b), M1 (Il1b and Il6) and M2 (Pgf and Mrc1) macrophage polarization markers in the macrophage cell line

Expression of *Kdm6a*, *Kdm6b*, *Il1b*, *Il6*, *Pgf* and *Mrc1* in mouse bone marrow-derived macrophages (a, c, e, g, i, k) and human peripheral blood-derived macrophages (b, d, f, h, j, l) non-polarized (M0) or polarized for 2 days either in pro-inflammatory macrophages (M1) with LPS (50 ng/ml) or immunomodulatory macrophages (M2) with a combination of both IL4 and IL10 (20 ng/ml) *in vitro* was measured by RT-qPCR. Data are presented as mean expression reported to M0 macrophages ± SEM with *B2M* serving as internal control of 6 independent mice (n=6) and 3 to 4 independent human healthy donors (n=3 for M1, n=4 for M0 and M2) in duplicate.

The p values are depicted as asterisks after Kruskal-Wallis test in the figures as follows: ***p <0.01; **p <0.01; *p <0.05 and ns: non-significant. Source data are provided as a Source Data file.

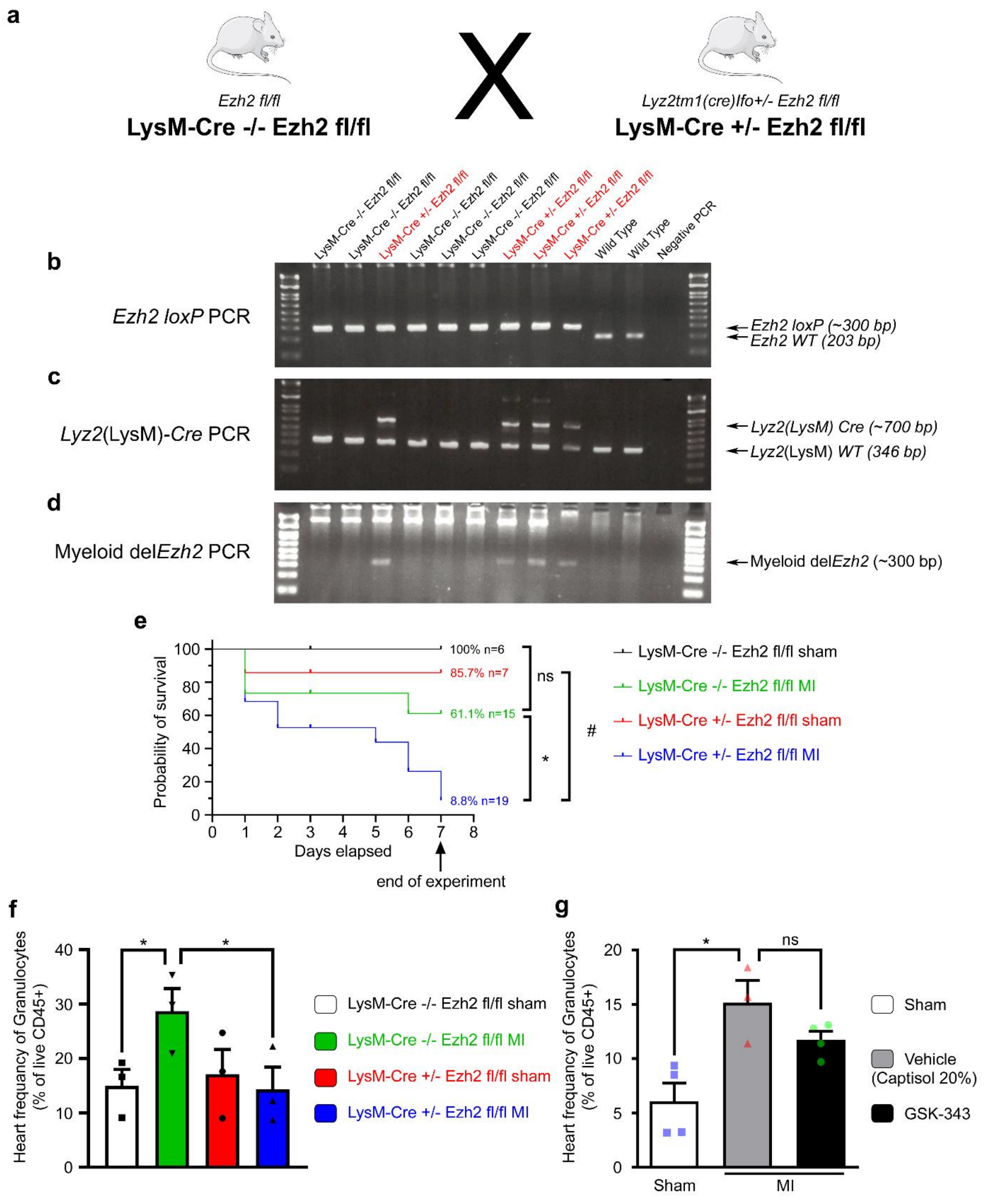

Figure S17: Myeloid-specific Ezh2 deletion impairs cardiac recovery after MI

Breeding scheme used to produce myeloid selective *Ezh2* deleted mice (LysM-Cre+/-Ezh2 fl/fl) (a) and representative pictures of *Ezh2 loxP* (b), *Lyz2*(LysM)-*Cre* (c) and *myeloid deleted (del) Ezh2* (d) genotyping PCR results. Kaplan–Meier survival curves

for littermate (LysM-Cre-/- Ezh2 fl/fl) or LysM-Cre+/- Ezh2 fl/fl mice after sham or permanent left coronary artery ligation-induced MI (e). The p values are depicted as asterisks (*) symbols for LysM-Cre+/- Ezh2 fl/fl MI versus LysM-Cre-/- Ezh2 fl/fl MI comparison or hashtag (#) symbols LysM-Cre+/- Ezh2 fl/fl MI versus LysM-Cre+/- Ezh2 fl/fl sham comparison after Mantel-Cox test in the figures as follows: * or # p <0.05; ns: non-significant. Cardiac granulocytes ($Cd11c^{neg}Cd11b^{hi}Ly6c^{med}Cx3cr1^{neg}$) population frequencies 3 days post-MI were evaluated by flow cytometry from 3 independent biological replicates (n=3) either sham or MI LysM-Cre-/- Ezh2 fl/fl or LysM-Cre+/- Ezh2 fl/fl mice (f) and sham (n=4) or MI mice treated daily either with vehicle (captisol 20%, n=3) or GSK-343 (n=4) (g). Data are expressed as mean frequency ± SEM of live $CD45^+$ cells. The p values are depicted as asterisks after Kruskal-Wallis test in the figures as follows: *p <0.05 and ns: non-significant. Source data are provided as a Source Data file. This Figure was partly generated using Servier Medical Art, provided by Servier, licensed under a Creative Commons Attribution 3.0 unported license.
