# Supplementary material for: Ezh2 emerges as an epigenetic checkpoint regulator during monocyte differentiation limiting cardiac dysfunction post-MI
Source: Nat Commun. 2023 Jul 25;14:4461. doi: 10.1038/s41467-023-40186-0 (PMC10368741; doi:10.1038/s41467-023-40186-0)
Supplement: Supplementary file 3 — Description of Additional Supplementary Files [file 41467_2023_40186_MOESM3_ESM.pdf]

Description of Additional Supplementary Files

Supplementary Data 1: List of bivalent genes identified by ChIP-Sequencing in CD14+ circulating human monocytes

Supplementary Data 2: List of GSK-343 down-regulated genes and related GO categories in selected circulating human monocytes

Down-regulated genes (combined data from three different donors, n = 3) with Benjamini-Hochberg adjusted p-value < 0.05 were considered significant. GO functional biological process category gene-enrichment was obtained from DAVID annotation terms after Fisher's Exact test.

Supplementary Data 3: List of GSK-343 up-regulated genes and related GO categories in selected circulating human monocytes

Up-regulated genes (combined data from three different donors, n = 3) with Benjamini-Hochberg adjusted p-value < 0.05 were considered significant. GO functional biological process category gene-enrichment was obtained from DAVID annotation terms after Fisher's Exact test.

Supplementary Data 4: Human RT-qPCR primer list

Supplementary Data 5: Mouse RT-qPCR primer list

Supplementary Data 6: ChIP-qPCR primer list
