# Supplementary material for: Ezh2 emerges as an epigenetic checkpoint regulator during monocyte differentiation limiting cardiac dysfunction post-MI
Source: Nat Commun. 2023 Jul 25;14:4461. doi: 10.1038/s41467-023-40186-0 (PMC10368741; doi:10.1038/s41467-023-40186-0)
Supplement: Supplementary file 10 — Reporting Summary [file 41467_2023_40186_MOESM10_ESM.pdf]

nature portfolio

Corresponding author(s): Sylvain Fraineau

Last updated by author(s): Jun 29, 2023

Reporting Summary

Nature Portfolio wishes to improve the reproducibility of the work that we publish. This form provides structure for consistency and transparency in reporting. For further information on Nature Portfolio policies, see our Editorial Policies and the Editorial Policy Checklist.

Statistics

For all statistical analyses, confirm that the following items are present in the figure legend, table legend, main text, or Methods section.

n/a	Confirmed	
☐	☒	The exact sample size (*n*) for each experimental group/condition, given as a discrete number and unit of measurement
☐	☒	A statement on whether measurements were taken from distinct samples or whether the same sample was measured repeatedly
☐	☒	The statistical test(s) used AND whether they are one- or two-sided<br>*Only common tests should be described solely by name; describe more complex techniques in the Methods section.*
☐	☒	A description of all covariates tested
☐	☒	A description of any assumptions or corrections, such as tests of normality and adjustment for multiple comparisons
☐	☒	A full description of the statistical parameters including central tendency (e.g. means) or other basic estimates (e.g. regression coefficient) AND variation (e.g. standard deviation) or associated estimates of uncertainty (e.g. confidence intervals)
☐	☒	For null hypothesis testing, the test statistic (e.g. *F*, *t*, *r*) with confidence intervals, effect sizes, degrees of freedom and *P* value noted<br>*Give P values as exact values whenever suitable.*
☒	☐	For Bayesian analysis, information on the choice of priors and Markov chain Monte Carlo settings
☒	☐	For hierarchical and complex designs, identification of the appropriate level for tests and full reporting of outcomes
☒	☐	Estimates of effect sizes (e.g. Cohen's *d*, Pearson's *r*), indicating how they were calculated

Our web collection on statistics for biologists contains articles on many of the points above.

Software and code

Policy information about availability of computer code

Data collection	No particular custom algorithms or software not yet described in published literature were used for data collection.
Data analysis	*Provide a description of all commercial, open source and custom code used to analyse the data in this study, specifying the version used OR state that no software was used.*

For manuscripts utilizing custom algorithms or software that are central to the research but not yet described in published literature, software must be made available to editors and reviewers. We strongly encourage code deposition in a community repository (e.g. GitHub). See the Nature Portfolio guidelines for submitting code & software for further information.

Data

Policy information about availability of data

All manuscripts must include a data availability statement. This statement should provide the following information, where applicable:

- Accession codes, unique identifiers, or web links for publicly available datasets
- A description of any restrictions on data availability
- For clinical datasets or third party data, please ensure that the statement adheres to our policy

RNA-Seq and ChIP-seq data have been deposited into the public GEO database respectively GSE165543 and GSE226811
Links: https://www.ncbi.nlm.nih.gov/geo/query/acc.cgi?acc=GSE165543 and https://www.ncbi.nlm.nih.gov/geo/query/acc.cgi?acc=GSE226811

Human research participants

Policy information about studies involving human research participants and Sex and Gender in Research.

Reporting on sex and gender	None of the findings in this article are relevant to patient sex and/or gender
Population characteristics	All information relevant to the patients characteristics are provided as supplementary information in supplementary tables 2 and 5
Recruitment	Between Nov 2018 and June 2020, the EPICAM prospective study enrolled 48 patients at Rouen University Hospital after diagnosis based on coronary angiography procedure. According to French legislation, all patients read the information note detailing the study protocol. All patients orally consented to the collection of an additional volume of blood during their usual care as well as the processing of their personal data (such as age, sex and coronary diagnosis) prior to the blood sampling. Inclusion criteria were: 1) age greater than or equal to 18 years and 2) admission to hospital for a coronary angiography. The exclusion criteria were 1) Infectious diseases, 2) Current pregnancy or breastfeeding, 3) Obesity (BMI > 30 kg/m²), 4) Hematological pathologies, 5) Anemia and 6) Inflammatory and autoimmune pathologies. As this is a monocentic study with a different number of patients included in each group, selection bias may be present and migth have impacted the results.
Ethics oversight	The CPP Ile de France V has approved the study august 10th 2018 (RCB: 2018-A02108-47).

Note that full information on the approval of the study protocol must also be provided in the manuscript.

Field-specific reporting

Please select the one below that is the best fit for your research. If you are not sure, read the appropriate sections before making your selection.

[x] Life sciences [ ] Behavioural & social sciences [ ] Ecological, evolutionary & environmental sciences

For a reference copy of the document with all sections, see nature.com/documents/nr-reporting-summary-flat.pdf

Life sciences study design

All studies must disclose on these points even when the disclosure is negative.

Sample size	Sample size were chosen according to the experimental experience of the procedures in the laboratory (PMID: 32404007, 27059805, 24760754). Regarding Human research participants, the sample size was determined by the duration of the recruitment period in agreement with the duration of the study approved by the CPP Ile de France V.
Data exclusions	Data were excluded based on the identification of outliers provided by GraphPad Prism 8 software
Replication	Reproducibility of experiment was assessed by reproducing at least 3 three times every single experiment
Randomization	For Human research study, participant were not randomized but separately included in the study in double blind manner. Randomization was not relevant to this study as patients were included in one of the three groups based on a diagnosis obtained after coronary angiography procedure. For mouse in vivo study, animals were randomized after surgical procedure before receiving the pharmacological treatment.
Blinding	For Human and mouse in vivo study, investigators were blinded during experimental and result analysis procedures. This blind procedure was based on an anonymous number or a micro-chip anonymous number attributed to Human participants or mouse included in the study respectively.

Reporting for specific materials, systems and methods

We require information from authors about some types of materials, experimental systems and methods used in many studies. Here, indicate whether each material, system or method listed is relevant to your study. If you are not sure if a list item applies to your research, read the appropriate section before selecting a response.

Materials & experimental systems

n/a	Involved in the study
☐	☒ Antibodies
☐	☒ Eukaryotic cell lines
☒	☐ Palaeontology and archaeology
☐	☒ Animals and other organisms
☐	☒ Clinical data
☒	☐ Dual use research of concern

Methods

n/a	Involved in the study
☐	☒ ChIP-seq
☐	☒ Flow cytometry
☒	☐ MRI-based neuroimaging

Antibodies

Antibodies used

The full list of antibodies used in the study is provided in the main manuscript as well as below and separated depending of the application and used at the indicated dilutions:

Immunohistochemistry: rat anti-CD11b (BD Pharmingen Cat#557395, 1:1500), rat anti-CD68 (eBiosicence Cat#14-0681, 1:800), goat anti-CD206 (Thermo Fisher Cat#PA5-46994, 1:1500), rabbit anti-EZH2 (D2C9, Cell Signaling Cat#5246, 1:1000), rat anti-iNOS (W16030C, Biolegend Cat#696802, 1:500), WGA-FITC (Interchim#FP-CE8070, 1:100), biotinylated rabbit anti-Lyve1 (eBioscience#13-0443, 1:400), biotinylated rat anti-CD31 (BD pharmingen#553371, 1:50); biotinylated rabbit anti-Cx3cr1 (BIOSS Cat#bs-1728R-Biotin, 1:100), rat anti-Ly6c (W16030C, Abcam Cat#ab15627, 1:400), rat anti-Trem2 (RM0139-5J46, Abcam Cat#ab86491, 1:100), rat anti-Cd86 (B87-2, eBiosicence Cat#14-0862-82, 1:100), goat anti-IL-1 beta /IL-1F2 (R&D Systems Cat#AF401-NA, 1:100) and rat anti-MHC Class II (I-A_I-E) (M5/114.15.2, eBiosicence Cat#14-5321-82, 1:200).

Chromatin Immunoprecipitation: 2 μg of the following antibodies were used: rabbit anti-H3K4me3 (Millipore, Cat#07-473), rabbit anti-H3K27me3 (Millipore, Cat#07-449), or normal rabbit IgG (Millipore, Cat#12-370).

Flow cytometry: anti-mouse CD45 (30F11)-BV711 (Biolegend Cat#103147, 1:200); anti-mouse CD11b (M1/70)-FITC (BD Pharmingen Cat#553310, 1:200); anti-mouse Ly6C (HK1-4)-APC/Cy7 (Sony Cat#1240130, 1:200); anti-mouse Cx3cr1 (SA011F11)-BV421 (Biolegend Cat# 149023, 1:200); anti-mouse CD86 (GL-1)-BV650 (Sony Cat#1125175, 1:200); anti-mouse CD3ε (17A2)-BV785 (Biolegend Cat#100355, 1:200); anti-mouse CD115 (AFS798)-PE (Sony Cat#1277530, 1:200); anti-mouse CD11c (N418)-PE/Texas Red (Sony Cat#1186740, 1:200); anti-mouse CD19 (6D5)-PE/Cy5 (Biolegend Cat#115509, 1:200); anti-mouse CD206 (C068C2)-APC (Sony Cat#1308540, 1:200); anti-mouse IA-IEk (MHCII) (M5/114.15.2)-Alexa Fluor 700 (Sony Cat#1138110, 1:200); anti-mouse F4-80(BM8)-BV605 (Sony Cat#1215665, 1:200), LIVE/DEAD Viability/Cytotoxicity-UV (Invitrogen Cat#L23105, 1/1000).

Western blotting: anti-pan histone H3 (Millipore, Cat#07-690, 1:100 000), anti-H3K27me3 (Millipore, Cat#07-449, 1:2000).

Validation

Specificity and Specificity and sensibility for each of the primary antibodies used for each application has been validated in the laboratory based on advised concentration or dilution provided by the manufacturer at first and refined using appropriate positive and negative controls relevant to each application. For instance anti-CD206 specificity and sensibility for Immunohisto/cytochemistry was assessed with cell autofluorescence control as well as cell negative control (cells from the same cell lineage not expressing CD206, i.e. M0 and M1 macrophages) and cell positive control (cells from the same cell lineage expressing CD206, i.e. M2 macrophages). Similar types of procedure were applied for flow cytometry (use of (use of IgG Isotype controls, compensation matrix, cell positive and negative controls), for western blotting (with additional sensitivity assay following sequential increased protein loading), and Chromatin Immunoprecipitation (use of IgG Isotype controls, background or signal level measurement on well-known negative and positive genomic regions respectively as described in Figures 3a, 3c and 5).for each of the primary antibodies used for each application has been validated in the laboratory based on advised concentration or dilution provided by the manufacturer at first and refined using appropriate positive and negative controls relevant to each application. For instance anti-CD206 specificity and sensibility for Immunohisto/cytochemistry was assessed with cell autofluorescence control as well as cell negative control (cells from the same cell lineage not expressing CD206, i.e. M0 and M1 macrophages) and cell positive control (cells from the same cell lineage expressing CD206, i.e. M2 macrophages).

Eukaryotic cell lines

Policy information about cell lines and Sex and Gender in Research

Cell line source(s)

Monocytes were directly or indirectly (after red blood cell lysis (eBioscience Cat#00-4333-57) isolated by negative selection using EasySep™ monocyte isolation kits for human (male and female, using StemCell Technologies Cat#19669) and mouse (female, using StemCell Technologies Cat#19861) cells from fresh peripheral blood samples according to the manufacturer's instructions.
TIB-204™ (WEHI-265.1) mouse monocyte cell line (ATCC Lot#4249478) was used in this study.

Authentication

Primary cells were authenticated following the expression of specific markers Cd11b+/Cd68- for monocytes, Cd68+/iNos-/Cd206- for M0 macrophages, Cd68+/iNos+/Cd206- for M1 macrophages and Cd68+/iNos-/Cd206+ for M2 macrophages (Fig. 2). TIB-204™ (WEHI-265.1) mouse monocyte cell line was authenticated with Cx3cr1 and Ly6c expression (Supplementary Fig. S9B). TIB-204 differentiation into M0 and polarization into M1 or M2 macrophages was assessed by RT-qPCR (Supplementary Fig. S13A).

Mycoplasma contamination

Cell lines were not tested for mycoplasma contamination

Commonly misidentified lines (See ICLAC register)	TIB-204™ (WEHI-265.1) have not been identified in the register

Animals and other research organisms

Policy information about studies involving animals; ARRIVE guidelines recommended for reporting animal research, and Sex and Gender in Research

Laboratory animals	C57BL/6JRj (Janvier Labs) or genetically modified (LysM-Cre+/- Ezh2fl/fl and LysM-Cre-/- Ezh2fl/fl) female 12 to 18 weeks (20-22g) mice were used.<br>LysM-Cre+/- Ezh2fl/fl were obtain from breeding of LysM-Cre+/- Ezh2WT/WT (B6.129P2-Lyz2tm1(cre)Ifo/J (The Jackson Laboratory Cat#004781)) and LysM-Cre-/- Ezh2fl/fl (B6;129S1-Ezh2tm2Sho/J (The Jackson Laboratory Cat#022616)).
Wild animals	No use of wild animals
Reporting on sex	We only used female mice for MI studies as they display lower mortality than male in the MI model, which help us reduce the numbers of animals included in our studies. In addition, females express both alleles of the H3K27me3 demethylase Kdm6a gene encoding the Utx protein.
Field-collected samples	No field collected samples were used in the study
Ethics oversight	All animal experiments performed in this study were approved by the regional ethics review board (CENOMEXA) and the french Ministère de l'Enseignement Supérieur et de la Recherche, in line with E.U and French legislation, referred as APAFIS #8157-2016121311094625-v5 Normandy, APAFIS #31897-2021111911125883 v4.

Note that full information on the approval of the study protocol must also be provided in the manuscript.

Clinical data

Policy information about clinical studies

All manuscripts should comply with the ICMJEguidelines for publication of clinical research and a completedCONSORT checklist must be included with all submissions.

Clinical trial registration	This is a non interventional protocol registered as RCB: 2018-A02108-47
Study protocol	This is an internal non interventional protocol to the University Hospital of Rouen
Data collection	Between Nov 2018 and June 2020, the EPICAM prospective study enrolled 48 patients at Rouen University Hospital. The CPP Ile de France V has approved the study august 10th 2018 (RCB: 2018-A02108-47). All patients read the information sheet and accepted to participate to the study before blood collection. Inclusion criteria were: 1) age greater than or equal to 18 years and 2) admission to hospital for a coronary angiography. The exclusion criteria were 1) Infectious diseases, 2) Current pregnancy or breastfeeding, 3) Obesity (BMI > 30 kg/m²), 4) Hematological pathologies, 5) Anemia and 6) Inflammatory and autoimmune pathologies.<br>During the coronarography procedure, peripheral blood was collected into 4 BD Vacutainer® EDTA K2 tubes (Becton Dickinson Cat#367862). In-hospital data were entered into a dedicated database.
Outcomes	The objective of this non interventional protocol was to study the whole transcriptomic profile human monocytes in patients assesed by mRNA-seq and RTqPCR methods.

ChIP-seq

Data deposition

- [x] Confirm that both raw and final processed data have been deposited in a public database such as GEO.
- [x] Confirm that you have deposited or provided access to graph files (e.g. BED files) for the called peaks.

Data access links<br>*May remain private before publication.*	We downloaded data sets from the ENCODE portal48 (https://www.encodeproject.org/) with the following identifiers: ENCSR267NWZ, and ENCSR000ASK respectively for human CD14+ monocytes H3K4me3 (GSM1003536) and H3K27me3 (GSM1003564) ChIP-seq data sets. Peak-calling for bivalent genes are under GSE226811
Files in database submission	ENCSR267NWZ : CD14+ monocytes H3K4me3 (GSM1003536)<br>ENCSR000ASK : CD14+ monocytes H3K27me3 (GSM1003564)<br>Bivalent Gene Peak Calling GSE226811
Genome browser session (e.g. UCSC)	https://genome.ucsc.edu/s/teetoonet/Human%20CD14%2B%20monocytes%20bivalent%20genes

Methodology

Replicates	ENCSR267NWZ : 1 biological and 2 technical replicates, https://www.encodeproject.org/files/ENCFF210FYU/<br>ENCSR000ASK : 2 biological and 1 technical replicates, https://www.encodeproject.org/files/ENCFF565AEM/
Sequencing depth	ENCSR267NWZ : https://www.encodeproject.org/files/ENCFF210FYU/

	ENCSR000ASK : https://www.encodeproject.org/files/ENCFF565AEM/
Antibodies	ENCSR267NWZ : H3K4me3<br>ENCSR000ASK : H3K27me3
Peak calling parameters	Peak calling was performed with MACS2 using default parameters for H3K4me3 samples and with the addition –borad option with H3K27me3 samples. Overlapping peaks were identified with the intersect Bed tool from the bedtools package version 2.26.
Data quality	Reads were aligned to the hg19 genome build using Bowtie2 version 2.3.4.149. Unmapped and duplicate reads were removed with Samtools version 1.5. Peak calling was performed with MACS2 using default parameters for H3K4me3 samples and with the addition –borad option with H3K27me3 samples. Peaks called by MACS2 with q-values < 0.05 were retained.
Software	Reads were aligned to the hg19 genome build using Bowtie2 version 2.3.4.149. Unmapped and duplicate reads were removed with Samtools version 1.5. Peak calling was performed with MACS2 using default parameters for H3K4me3 samples and with the addition –borad option with H3K27me3 samples. Overlapping peaks were identified with the intersect Bed tool from the bedtools package version 2.26. Annotation of common H3K4me3 and H3K27me3 peaks was performed with PAVIS (https://manticore.niehs.nih.gov/pavis2/).

Flow Cytometry

Plots

Confirm that:

- [x] The axis labels state the marker and fluorochrome used (e.g. CD4-FITC).
- [x] The axis scales are clearly visible. Include numbers along axes only for bottom left plot of group (a 'group' is an analysis of identical markers).
- [x] All plots are contour plots with outliers or pseudocolor plots.
- [x] A numerical value for number of cells or percentage (with statistics) is provided.

Methodology

Sample preparation	Cardiac samples were collected at 3 or 8 days post-MI. Left ventricles (LV) were harvested after perfusion with physiologic serum at 37°C. Infarcted Scar and Border Zones (BZ) were carefully separated and collected in 50ml-falcon tube containing 5mL of RPMI 1640 medium. Three LVs from same group were collected per tube. Digestion enzymes cocktail (collagenase II (5 mg), Dispase (6 mg), DNAse I (300 µg) (Sigma) per 5mL) was added and sample were dissociated through GentleMACSTM (Miltenyi) for 15min. After dissociation, samples were filtered through 70 µm and then 40 µm cell strainer and prepared for FACS staining in PBS-2% FBS buffer.
Instrument	Data were acquired with LSRFortessa Cell Analyzer (BD Bioscience)
Software	Data were analyzed using FlowJo v10.8.1
Cell population abundance	We did not perform flow cytometry sorting in this study
Gating strategy	CD45+ leucocytes gating strategy was used to exclude endothelial cells and other cardiac cell types (Supplementary Fig. S14). On live cells, lymphocytes were gated out by CD3 or CD19 staining. Then, dendritic cells were excluded from the analysis by gating out CD11c+ cells. In the CD11b+CD11c- pool, monocyte/macrophage cell populations were analyzed based on their expression of Ly6C versus Cx3cr1. Granulocytes were excluded by their lower expression of Cx3cr1 and higher Side Scatter (SSC) profile compared to monocytes/macrophages subset. Classical/inflammatory and non-classical monocytes/ macrophages were defined as Ly6Chi/Cx3cr1hi and Ly6Clo/Cx3cr1hi, respectively (Supplementary Fig. S14).

- [x] Tick this box to confirm that a figure exemplifying the gating strategy is provided in the Supplementary Information.
